# Long-term effects of ruxolitinib versus best available therapy on bone marrow fibrosis in patients with myelofibrosis

**DOI:** 10.1186/s13045-018-0585-5

**Published:** 2018-03-15

**Authors:** Hans Michael Kvasnicka, Jürgen Thiele, Carlos E. Bueso-Ramos, William Sun, Jorge Cortes, Hagop M. Kantarjian, Srdan Verstovsek

**Affiliations:** 1 Senckenberg Institute of Pathology, University of Frankfurt, Theodor-Stern-Kai 7, 60590 Frankfurt, Germany; 2 University of Cologne, Cologne, Germany; 3 Department of Hematopathology, The University of Texas MD Anderson Cancer Center, Houston, TX USA; 4 Incyte Corporation, Wilmington, DE USA; 5 Department of Leukemia, The University of Texas MD Anderson Cancer Center, Houston, TX USA

**Keywords:** Bone marrow fibrosis, Myelofibrosis, Ruxolitinib, Hydroxyurea

## Abstract

**Background:**

Myelofibrosis (MF) is a life-shortening complication of myeloproliferative neoplasms associated with ineffective hematopoiesis, splenomegaly, and progressive bone marrow (BM) fibrosis. The oral Janus kinase (JAK) 1/JAK2 inhibitor ruxolitinib has been shown to improve splenomegaly, symptom burden, and overall survival in patients with intermediate-2 or high-risk MF compared with placebo or best available therapy (BAT).

**Methods:**

The effects of ruxolitinib therapy for up to 66 months on BM morphology in 68 patients with advanced MF with variable BM fibrosis grade were compared with those in 192 matching patients treated with BAT. Available trephine biopsies underwent independent, blinded review by three hematopathologists for consensus-based adjudication of grades for reticulin fibrosis, collagen deposition, and osteosclerosis.

**Results:**

Ruxolitinib treatment versus BAT was associated with greater odds of BM fibrosis improvement or stabilization and decreased odds of BM fibrosis worsening based on changes from baseline in reticulin fibrosis grade. Generally, these changes were accompanied by a sustained higher level of individual spleen size reduction and regression of leukoerythroblastosis. Patients with more advanced baseline fibrosis showed lower spleen size response.

**Conclusions:**

The finding that long-term ruxolitinib therapy may reverse or markedly delay BM fibrosis progression in advanced MF suggests that sustained JAK inhibition may be disease-modifying.

**Trial registration:**

INCB18424-251, ClinicalTrials.gov identifier NCT00509899.

**Electronic supplementary material:**

The online version of this article (10.1186/s13045-018-0585-5) contains supplementary material, which is available to authorized users.

## Background

From a histopathology perspective, bone marrow (BM) fibrosis implies a process whereby increases in fibrous matrix are observed within the BM without explicit reference to quantity or quality (reticulin vs collagen); this can be caused by a variety of reactive as well as neoplastic disorders [1]. Generalization of the disease process in the course of *BCR-ABL1*-negative myeloproliferative neoplasms (MPNs) is usually associated with the development of BM fibrosis and ineffective hematopoiesis [2]. Along these lines, the prognostic value of BM fibrosis remains to be defined; however, several studies have provided emerging evidence that progression of BM fibrosis has a significant prognostic implication [3–12]. Myelofibrosis (MF) as a serious, life span-shortening complication of MPNs can present as primary MF (PMF) or develop secondary to polycythemia vera (post-PV MF) or essential thrombocythemia (post-ET MF) [2, 13]. The clinical presentation of individual patients is highly heterogeneous [2], and the prognosis at diagnosis may vary greatly [14–16]. Recently, BM fibrosis has been reported to be an independent negative prognostic factor in PMF [5, 8, 10, 17].

The initial emergence and time-dependent progression of BM fibrosis in patients with MPN-associated MF is believed to be a concomitant effect of neoplastic (clonal) myeloproliferation [18, 19]. This assumption is supported by clinical cases of relatively rapid regression of BM fibrosis after allogeneic hematopoietic stem cell transplantation [20–22] and by evidence from preclinical models that suggest BM fibrosis may be mediated by clonal cell-derived cytokines and inflammatory stromal reactive pathobiological changes [23, 24]. However, the precise role of BM fibrosis in MPN disease progression remains incompletely understood. This is in part due to the difficulties in evaluating corresponding changes in BM histopathology by means of standardized sequential biopsy examinations and the consequent paucity of relevant clinical data [25, 26]. Moreover, although consensus guidelines establishing a simplified scoring system for reticulin fibrosis in MPN have been developed [27] and were adopted by the World Health Organization (WHO) [13], comparable guidelines for the evaluation of the equally important processes of collagen deposition and osteosclerosis have been developed only recently [25].

Major unresolved questions in MPNs are the prognostic implications of BM fibrosis grade at diagnosis and the relationship between BM fibrosis and clinical status, including the clinical impact of fiber regression. Results of former investigations [4–6, 17, 27], and particularly recent studies in patients with PMF, strongly suggest that WHO-defined BM fibrosis grade may have prognostic value independent of International Prognostic Scoring System (IPSS) risk stratification [8–11]. Furthermore, in patients with MPN-associated MF, higher BM fibrosis grades are associated with worsening clinicohematologic manifestations, such as lower levels of hemoglobin, higher percentage of peripheral blasts, increased splenomegaly, and higher IPSS and Dynamic IPSS risk scores [8–11, 28]. Except for a few patients with very early stages of PMF [29, 30] or post-PV MF [31], conventional treatment, including a variety of cytoreductive agents, has not been shown to consistently result in the resolution of BM fibrosis [7, 32].

The highly selective oral Janus kinase (JAK) 1/JAK2 inhibitor ruxolitinib has been shown in two phase 3 studies to reduce splenomegaly and symptom burden in patients with intermediate-2 or high-risk disease compared with placebo or best available therapy (BAT) [33–37] with concomitant improvements in functional status and various measures of quality of life [38, 39]. Moreover, longer-term data from the phase 3 COMFORT (COntrolled MyeloFibrosis study with ORal JAK inhibitor Treatment) trials showed that ruxolitinib was associated with a survival advantage compared with the respective controls in each study [35–37, 40–42]. Despite the rapid clinical improvements observed in the vast majority of patients treated with JAK-inhibitors like ruxolitinib, short-term local assessments (e.g., ≤ 6 months of therapy) did not reveal any substantial treatment impact on BM histomorphology [33, 42–45]. Therefore, these findings raise the possibility that long-term therapy with ruxolitinib may influence the evolution of disease progression [46].

To further explore this possibility, we evaluated the effects of long-term ruxolitinib therapy on BM fibrosis in patients enrolled in the aforementioned phase 1/2 study at the MD Anderson Cancer Center (MDACC) and compared them with changes in BM fibrosis observed in a control cohort of patients with PMF and matching degrees of BM fibrosis at baseline who received conventional (best available) therapy.

## Methods

### Patients

The ruxolitinib cohort used for this analysis included patients with PMF, post-PV MF, or post-ET MF enrolled in a phase 1/2, single-arm, open-label study (INCB18424-251, NCT00509899) [47]. Patients eligible for this analysis were enrolled at the MDACC and had available trephine biopsy data at both baseline and 24 months of therapy. Median disease duration before study inclusion was 73 months (0–370 months). For many patients, BM biopsies were obtained up to 66 months after initiation of ruxolitinib treatment. Patients presented with intermediate- or high-risk disease (by the Lille scoring system) [48], marked splenomegaly (i.e., with spleen length palpable ≥ 10 cm below the left costal margin), and required therapy. The median treatment duration was 30.5 months (24–72 months), and the median follow-up time was 52 months (24–76 months).

The control cohort for this analysis consisted of 192 patients with PMF who received BAT. BAT included hydroxyurea (hydroxycarbamide; 45%), interferon-α (various formulations, including PEGylated forms; 8%), assorted sequential therapies (25%), and supportive-only therapy (22%). The patients for this cohort were identified in a large, independent, multicenter, and observational database and selected retrospectively primarily based on BM morphology, in particular baseline degree of BM fibrosis according to the WHO grading system [13, 25, 27] and follow-up time to match with the ruxolitinib cohort. In the BAT cohort, the majority of baseline biopsies were performed at disease onset (disease duration 0–6 months), while about 85% of the sequential trephine biopsies were done prospectively following either routine institutional practice or clinical protocols in the many participating university centers. In contrast, BM trephines were performed in the ruxolitinib cohort according to the study protocol [47, 49]. Only in 15% of the BAT group a change in clinical presentation (i.e., switch from IPSS intermediate 1 to intermediate 2, increase in blast percentage, etc.) would have prompted a repeated BM biopsy evaluation. The median treatment duration was 38 months (24–68 months), and the median follow-up time was 45 months (24–98 months).

### Assessment of bone marrow morphology

Sections of formalin-fixed and paraffin-embedded BM trephine biopsies were stained with silver impregnation following Gordon-Sweets’ method, Masson trichrome staining, and hematoxylin-eosin to assess reticulin fibers, amount of collagen deposition, and degree of osteosclerosis, respectively.

Grading of BM fibrosis grade was based on the WHO grading system including the new grading systems for the degrees of collagen deposition and osteosclerosis [13, 25, 27]. Samples from the BAT cohort were evaluated for WHO-defined BM fibrosis grade only [13, 25, 27]. Moreover, in the samples from the MDACC ruxolitinib-treated cohort, changes of hematopoietic cellularity following therapy were evaluated. Three hematopathologists (HMK, JT, and CEB-R) reviewed all study samples in an independent assessment with final grading based on consensus. Grading accuracy was subsequently validated in randomly selected samples by a consortium of eight hematopathologists from the European Leukemia Net (ELN) Consensus experts group.

### Statistical analyses

A Cochran-Mantel-Haenszel test for ranked data was applied to validate uniformity of BM fibrosis grade distribution at baseline between the ruxolitinib and BAT cohort. Changes in BM fibrosis grades, collagen deposition, and grade of osteosclerosis at various time points versus baseline were categorized for each patient as individual improvement (decrease), stabilization (no change), or worsening (increase). Because patients with baseline BM fibrosis grade 3 cannot further progress by definition, these cases have been excluded in the subgroup analysis for worsening. Therefore, the denominator for this analysis is different from the improvement and/or stabilization group (see Table 2 and Fig. 2). Odds ratios with corresponding 95% confidence intervals for worsening, improvement, and improvement or stabilization in BM fibrosis grade were determined by logistic regression analysis controlled for baseline fibrosis grade. This study was not intended to be a formal comparison between ruxolitinib and BAT-treated cohorts for a variety of statistical reasons. In particular, a statistical comparison with regard to BM fibrosis was not part of the study protocol. Furthermore, the selection of patients was different between both the ruxolitinib and BAT groups (prospective trial versus observational control group). Therefore, we have provided only corresponding odds ratios to emphasize this difference and not to overestimate or over-interpret our results.

Individual changes in hematopoietic cellularity at different time points following ruxolitinib therapy were calculated as relative change to the expected age-matched amount [27, 50]. In this regard, a value of 1.0 indicated a normal amount of hematopoietic tissue as seen in a healthy individual, while values less than 1.0 represented a therapy-induced decrease and values above 1.0 were in line with increased cellularity probably reflecting loss of myeloproliferative control. Therapy-related spleen size reduction was assessed in the ruxolitinib cohort according to the recently published International Working Group-Myeloproliferative Neoplasms Research and Treatment (IWG-MRT) response criteria [51]. Briefly, a relative decrease in palpable spleen length below the left costal margin of more than 50% was required to qualify for spleen response.

## Results

### Patients

At the time of analysis for this project, 68 of the 107 patients enrolled at the MDACC in the ruxolitinib phase 1/2 study had BM data at baseline and 24 months of therapy; 38, 23, 10, and 4 patients also had BM data at months 48, 54, 60, and 66, respectively. Reasons for study drop-off in the ruxolitinib cohort included loss of clinical response or progressive disease (*n* = 11), and on-study death from unrelated causes (*n* = 11). Of the 68 subjects, 36 patients were still on study by the time of data cut-off. Regarding the BAT control group, BM biopsies from 192 patients with PMF were performed after 24 (98 patients), 48 (65 patients), 54 (16 patients), 60 (9 patients), or 66 (6 patients) months. Patients’ demographic and clinical characteristics at baseline as well as BM fibrosis grading are shown for each cohort in Table 1. Statistical analysis confirmed a uniform percentage distribution of baseline BM fibrosis grades between the ruxolitinib and BAT cohorts (*P* = 0.3298; Cochran-Mantel-Haenszel test); furthermore, there was no statistical difference for individual baseline fibrosis grades between both groups (*P* = 0.098; two-sided Pearson’s chi-squared test). However, patients in the BAT group, who were matched to the ruxolitinib cohort, had lower IPSS scores, which was also reflected by less severe splenomegaly and higher hemoglobin values and platelet counts at baseline (Table 1). Consequently, the proportion of grade 1 baseline BM fibrosis was slightly higher in the BAT group. However, the primary aim of this analysis was to identify adequate numbers of cases with matched BM biopsy data (rather than clinical characteristics) receiving BAT for all given time endpoints as defined by the ruxolitinib study protocol (24, 48, and 60 months) [47, 49]. With regard to the overall unfavorable prognosis reported in the BAT cohort [2, 14, 16, 48, 52], a higher proportion of lower IPSS risk patients reaching these endpoints had to be expected.

**Table 1:** Baseline patient and disease characteristics

Characteristic	Ruxolitinib (*n* = 68)	BAT (*n* = 192)	*p*
Demographics			
Mean age (95% CI), years	66.8 (65.1 to 68.5)	59.1 (57.7 to 61.3)	< 0.001
Male sex, %	57	48	0.205
Clinical parameters			
IPSS risk status, %			0.001
High risk	57	15	
Intermediate-2	31	16	
Intermediate-1	12	39	
Low risk	0	30	
Mean spleen size (95% CI), cm^a^	19.6 (18.4 to 21.6)	3.6 (3.0 to 4.2)	0.001
Mean hemoglobin (95% CI), g/dL	10.8 (10.3 to 11.3)	12.1 (11.7 to 12.5)	0.001
Mean platelet count (95% CI), × 10^9^/L	401.4 (341.0 to 462.9)	521.1 (464.6 to 577.5)	0.015
Mean WBC count (95% CI), ×10^9^/L	17.7 (14.2 to 21.3)	13.4 (11.6 to 15.3)	0.021
Mean peripheral blasts (95% CI), %	0.8 (0.5 to 1.1)	0.5 (0.3 to 0.7)	0.097
WHO grade of BM fibrosis^b^, %			
Grade 1	22	37	0.098
Grade 2	53	52	
Grade 3	25	11	

*BAT* best available therapy, *CI* confidence interval, *IPSS* International Prognostic Scoring System, *WBC* white blood cell, *BM* bone marrow

^a^Palpable spleen length below costal margin

^b^BAT patients with ≥ 60 months follow-up during study

At baseline, 78% of the patients in the ruxolitinib cohort presented with overt BM fibrosis (grade 2 or 3). Accumulation of collagen fibers was observed in 32 cases (47%), and half of the patients presented at baseline with osteosclerosis of various degrees. In the ruxolitinib cohort, the proportion of patients with grade 3 BM fibrosis at baseline was greater among those with high-risk status (30%) according to the IPSS score than those with intermediate-1 (11%) or intermediate-2 risk (21%). At baseline, wide ranges of age-adjusted hematopoietic cellularity were observed across all fiber grades (0.28–5.0); however, median values indicated an overall increase in cellularity (median value 2.4–2.7). A low cellularity index at baseline was associated in most cases with previous cytoreductive therapies before study entry.

Palpable spleen lengths at baseline in ruxolitinib-treated patients were greater among those with BM fibrosis grade 3 (median, 22.0; range, 11.0 to 35.0 cm), as compared with those of the patients presenting with grade 1 (median, 20.5; range, 11 to 29.0 cm) or grade 2 (median, 18.5; range, 11 to 29.0 cm).

### Treatment effects on bone marrow fibrosis

**Fig. 1:**
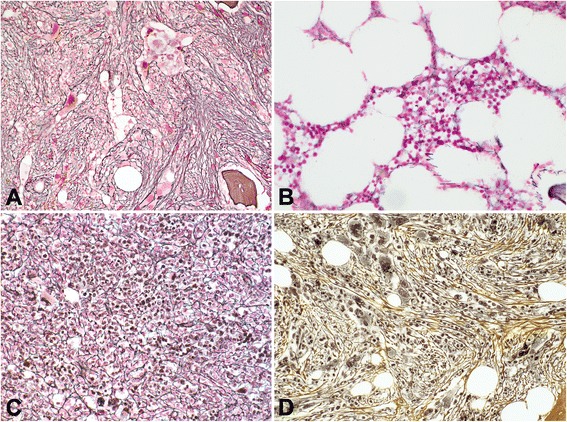
Changes in bone marrow (BM) fibrosis at 48 months following ruxolitinib (**a**, **b**) and hydroxyurea (**c**, **d**) therapy. Ruxolitinib therapy induced a significant regression of BM fibrosis from baseline grade 3 (**a**) to grade 0 (**b**). Hydroxyurea treatment had no impact on reversal of BM fibrosis; biopsy at baseline revealed a grade 1 (**c**), and 48 months trephine showed an increase in reticulin to grade 2 (**d**)

**Fig. 2:**
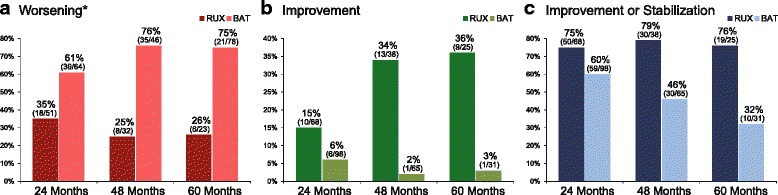
Changes in bone marrow fibrosis grade during ruxolitinib (RUX) therapy and best available therapy (BAT). Individual changes were calculated as **a** worsening (cases with baseline BM fibrosis grade 3 were excluded from this analysis because further progression is not defined), **b** improvement, or **c** improvement or stabilization

Example of one patient with changes in BM reticulin fibrosis associated with ruxolitinib therapy as contrasted to those seen with BAT are shown in Fig. 1a–d. Worsening of BM fibrosis was more common with BAT than ruxolitinib treatment (Fig. 2a). In patients with baseline BM fibrosis grade 1 or 2, the odds ratio (Table 2) for an increase in grading at 24, 48, and 60 months of therapy were lower with ruxolitinib therapy than BAT (Fig. 2a, Table 2). Conversely, ruxolitinib-treated patients with grades 1 to 3 revealed a higher odds ratio for achieving improvement or stabilization of BM fibrosis (Fig. 2b, c). In the ruxolitinib cohort, improvement or stabilization of BM fibrosis at 24, 48, and 60 months of therapy generally was accompanied by improvement or stabilization of collagen deposition and osteosclerosis (Additional file 1: Figure S1A to C). All ruxolitinib-treated patients demonstrated a linear-monotone pattern of change over time in BM fibrosis grade. None of these cases showed initially an improvement and then a subsequent worsening. However, a significant number of patients revealed a stabilization at 24 months (40/68, 59%), which was followed by a regression of BM fibrosis thereafter, either at 48 months (7/21, 33%) or 60 months (7/14, 50%). According to the current WHO definition, the overall BM fibrosis grading is regarded as the biologically most important parameter; however, collagen and osteosclerosis are clearly linked with this feature as higher WHO BM fibrosis grade in most cases is associated with higher amount of collagen and advanced osteosclerosis. Accordingly, cases with stabilization or improvement of collagen showed in general the same positive effect for the overall WHO BM fibrosis grading. On the other side, progression of BM morphology, i.e., worsening of fibrosis, was closely related to increase of osteosclerosis (Additional file 1: Figure S1).

**Table 2:** Odds ratio and 95% CI for BM fibrosis change according to therapy (ruxolitinib vs BAT)

BM fibrosis	Ruxolitinib vs BAT, *n*	Odds ratio^†^	95% CI
Worsening*			
24 months	18/51* vs 39/64*	0.38	0.17–0.84
48 months	8/32* vs 35/46*	0.11	0.01–0.32
60 months	6/23* vs 21/78*	0.07	0.01–0.34^‡^
Improvement**			
24 months	10/6 vs 6/98	3.10	1.01–9.50
48 months	13/38 vs 13/65	99.05	8.47–> 999
60 months	9/25 vs 1/31	19.29	2.13–174.89
Improvement or stabilization**			
24 months	50/68 vs 59/98	2.62	1.20–5.73
48 months	30/38 vs 30/65	9.40	3.18–27.79
60 months	19/25 vs10/31	15.39	2.97–79.67^‡^

*CI* confidence interval

*Patients with baseline BM fibrosis grade 3 were excluded from this analysis because further progression is not defined. An odds ratio < 1.0 favors ruxolitinib over BAT

**An odds ratio > 1.0 favors ruxolitinib over BAT

^†^Odds ratio determined by logistic regression controlled for baseline BM fibrosis grade

^‡^The last available grade from 54, 60, or 66 months was used

To exclude a potential bias generated by a study drop-off in the ruxolitinib cohort, we performed a stratified sub-analysis that compared patients still on study by the time of data cut-off with cases that dropped off after 24 or 48 months. There was no statistical difference regarding BM response between both groups (two-sided Pearson’s chi-squared test: 24 months *P* = 0.674; 48 months *P* = 0.551). Improvement in BM fibrosis at 24 months was seen in 11.1% of patients still on study as compared to 18.8% in the group of cases that later dropped off. Frequency of stabilization or progression was similar in both patient groups (stabilization 61.1 vs 56.2%; progression 27.8 vs 25.0%). At 48 months, similar findings were found; more than 30% of patients in both groups revealed a BM response (on study 34.5% vs study drop-off 33.3%). Similar results were obtained for stabilization or progression.

**Fig. 3:**
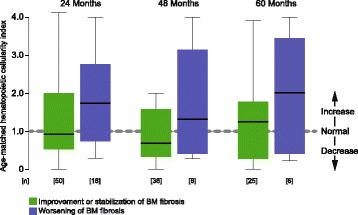
Changes in age-matched hematopoietic cellularity at different time points following ruxolitinib therapy. Values less than 1.0 indicate decreased cellularity, 1.0 reflects normal marrow cellularity, while values greater than 1.0 indicate increased cellularity

Normalization of age-adjusted hematopoietic cellularity following ruxolitinib treatment was in most cases associated with an improvement or stabilization of BM fibrosis at all time points (Fig. 3). Although a wide range of cellularity was calculable, patients with progressive BM fibrosis revealed a higher index implying a limited control of myeloproliferation.

**Fig. 4:**
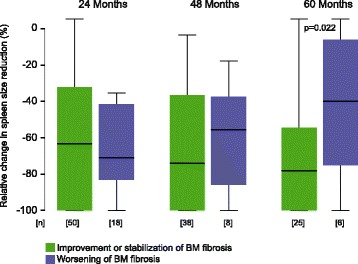
Relative change in palpable spleen size reduction from baseline at different time points following ruxolitinib therapy

**Fig. 5:**
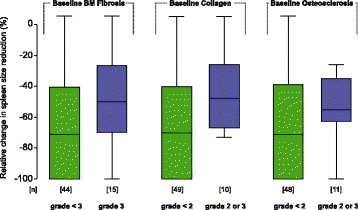
Relative change in palpable spleen size reduction following ruxolitinib therapy at month 24 according to baseline BM characteristics

Following long-term ruxolitinib therapy at 48 and 60 months, patients with improvement or stabilization of BM fibrosis achieved a higher level of individual spleen size reduction (Fig. 4). In keeping with this finding, response in palpable spleen size from baseline (≥ 50%) according to the revised response criteria for MF [51] showed a similar association with BM fibrosis. At 60 months of ruxolitinib therapy, 85% of patients with improvement or stabilization of BM fibrosis revealed a ≥ 50% spleen size response (24 months 72%, 48 months 80%) contrasting only 50% in the group with worsening of BM morphology. Along these lines, patients presenting with lower grades of baseline BM fibrosis, collagen, or osteosclerosis showed in general a higher degree of spleen size response (Fig. 5).

Furthermore, regression of leukoerythroblastosis following ruxolitinib therapy was associated with changes in BM fibrosis. In patients with improvement or stabilization of BM fibrosis in 77 and 83%, a durable reduction of circulating blasts to normal levels was evident at 24 and 48 months, respectively.

## Discussion

The results of this exploratory analysis suggest that long-term ruxolitinib therapy may delay or reverse BM fibrosis progression in patients with intermediate- or high-risk MPNs associated with MF across all aspects of the fibrotic process (i.e., reticulin fibrosis, collagen deposition, osteosclerosis). In this context, quantification and reading of collagen and osteosclerosis is important because of a possible therapy-related delinking between these BM stromal components as has been described following BM transplantation [20, 25]. Therefore, a separate assessment of these features certainly reflects therapeutic efficacy in a much appropriate way. Patients receiving ruxolitinib therapy had a significantly higher probability of improvement or stabilization of reticulin fibrosis, as compared with patients treated with BAT who were matched for BM morphology at baseline. In addition, ruxolitinib therapy was associated with a smaller likelihood of worsening BM fibrosis. Even though a higher number of lower risk (intermediate-1 and low-risk per IPSS) cases was included in the BAT cohort that also presented with smaller spleen sizes at study baseline, the observed differences to the ruxolitinib-treated group which comprised predominately of higher risk patients are even more striking. According to data available from the original IPSS-defining studies [14] recently provided evidence [5] and cumulative experience in clinical practice [9, 15, 48], the risk of progression in lower risk cases receiving BAT is expected to be significantly lower. Consequently, it can be assumed that our BAT cohort, which was derived from a large multicenter observational study, would also exhibit a similarly lower risk of progression. Noteworthy, decrease or stabilization in reticulin fibrosis grade was generally matched by concomitant changes in collagen deposition and osteosclerosis. In addition, durable improvement in splenomegaly was associated with a corresponding improvement or stabilization of BM morphology.

Stabilization or improvement of MPN-associated BM fibrosis in individual patients has also been observed in smaller patient subgroups treated with the JAK2 inhibitor fedratinib [43, 53]. In a total of 21 analyzed cases, stabilization or improvement of BM fibrosis from baseline has been reported in 44 to 83% at different time points [43, 53].

Taken together, these results suggest that long-term therapy with JAK inhibitors may exert a disease-modifying effect. It is tempting to speculate that the cellular components of the BM microenvironment that are linked to the fibrotic process are modulated by JAK inhibition. This observation would be compatible with the potential of this class of agents to reduce the inflammatory BM stromal reaction generally associated with MF [54]. With the exception of interferon, this type of effect has not been observed with conventional drugs, such as hydroxyurea.

This is the first extensive formal analysis of ruxolitinib effects on BM morphology using a consensus-based central review for grading of the fibrous matrix in patients with MPN-associated MF. This is an important difference to the recently published long-term COMFORT-II results, where evaluation of BM fibrosis was performed only by local pathologists at different (not standardized) time points in the ruxolitinib and BAT arm [42]. However, analysis of the published morphological data revealed an improvement of BM fibrosis in 24% of patients treated with ruxolitinib (median treatment duration, 2.2 years), while a similar effect was observed in 2.1% of cases in the BAT group (median treatment duration < 1 year). Comparable differences have been reported for stabilization of BM fibrosis between the ruxolitinib and BAT group at their last assessment during randomized treatment. Interestingly, the long-term results presented here indicate much higher rates of BM fibrosis improvement and/or stabilization beyond 24 months (Fig. 2 and Table 2). In particular, the percentage of patients in the ruxolitinib group showing biologically relevant BM fibrosis improvement increased from 15% at 24 months to 34 and 36% at months 48 and 60, respectively. Conversely, the rates of improvement in the BAT group dropped down from 6% to less than 3% in the 48 and 60 months subgroup analysis. It is important to emphasize that our findings following ruxolitinib therapy (Fig. 2 and Table 2) were not skewed by study drop-off or clinical response rates, because a specific subgroup analysis between patients still on study and those who dropped off at the different time points (i.e., 24, 48, and 60 months) did not reveal any difference in improvement rates. Considering these results, it can be speculated that treatment associated reconstitution and normalization of BM morphology in advanced high-risk MF patients takes longer than 2 years in most cases and that along these lines long-term therapy with ruxolitinib might induce some aspects of disease modification in responding patients.

Altogether, our data significantly extend previous findings, since we also applied the new WHO recommendations for the grading of BM morphology in patients with MF on active therapy [13]. These new guidelines incorporate specific grading recommendations for the quantification of not only the degree of BM reticulin fibrosis but also the degree of collagen deposition and osteosclerosis. By combining the WHO grading system for BM fibrosis with the straightforward proposals for scoring of collagen deposition and osteosclerosis together with a double review reading as a control mechanism, we could significantly minimize any unintended impairment of objectivity. In this regard, controversy and discussion persist regarding representativity of BM trephine biopsies as well as subjectivity and inter-observer variability with respect to fiber grading by various experts using different scoring systems [55]. Previous data suggest that myelofibrotic and osteosclerotic changes reveal a very uniform pattern throughout the whole skeleton [56] and therefore can be representatively assessed by a BM trephine biopsy. Furthermore, recent data have validated the high reliability of the applied WHO reticulin fibrosis grading system [26] as well as a strong reproducibility of the used grading for collagen and osteosclerosis [25]. It has been emphasized that a central review of BM fibrosis grading is mandatory in the context of clinical studies because of a more than 50% discrepancy with corresponding local evaluations [57]. Other studies have employed a computer-assisted image analysis for quantification of BM fibrosis and osteosclerosis [58]. This technique has been proposed as an objective method for quantification [58]. In our view, however, these computer-assisted scoring systems face technical problems similar to the established semi-quantitative grading methods. The objective numerical quantification of reticulin staining was defined as equivalent to the average percentage of black pixels in a digitized slide. Both the quality of the section and the staining [25], as well as the limitation to only three randomly selected hematopoietic areas (total area of 1.5 mm^2^) [58] may generate a significant bias. These impairments may be especially evident in patchy distributions of BM fibrosis throughout the marrow space following therapy [7, 25, 32]. On the other hand, it is noteworthy that the results of our analysis were subsequently randomly controlled and validated by a consortium of expert hematopathologists within an ELN collaborative project.

## Conclusions

The results of this exploratory analysis demonstrate that long-term ruxolitinib therapy may provide a marked delay or reversal in the progression of BM fibrosis in a large proportion of patients with MPN-associated MF. Taken together, our findings suggest sustained JAK1/JAK2 inhibition may be disease-modifying in these malignancies.

## Additional file

Additional file 1: **Figure S1.** Changes in bone marrow reticulin fibrosis, collagen deposition, and osteosclerosis in individual patients on ruxolitinib therapy. (A) 24, (B) 48, and (C) 60 months. (PDF 180 kb)

## Abbreviations

BAT: Best available therapy
BM: Bone marrow
ELN: European Leukemia Net
IPSS: International Prognostic Scoring System
IWG-MRT: International Working Group-Myeloproliferative Neoplasms Research and Treatment
JAK: Janus kinase
MF: Myelofibrosis
MPNs: Myeloproliferative neoplasms
PMF: Primary myelofibrosis
post-ET MF: Post-polycythemia vera myelofibrosis
post-PV MF: Post-essential thrombocythemia myelofibrosis
RUX: Ruxolitinib
WHO: World Health Organization
