# Supplementary figures and images for: Long-term effects of ruxolitinib versus best available therapy on bone marrow fibrosis in patients with myelofibrosis
Source: J Hematol Oncol. 2018 Mar 15;11:42. doi: 10.1186/s13045-018-0585-5 (PMC5856218; doi:10.1186/s13045-018-0585-5)

A. 24 Months

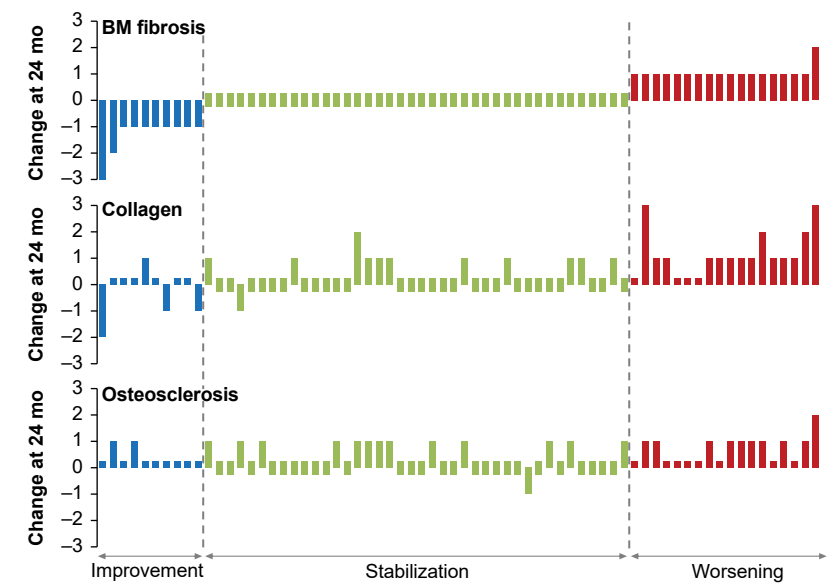

B. 48 Months

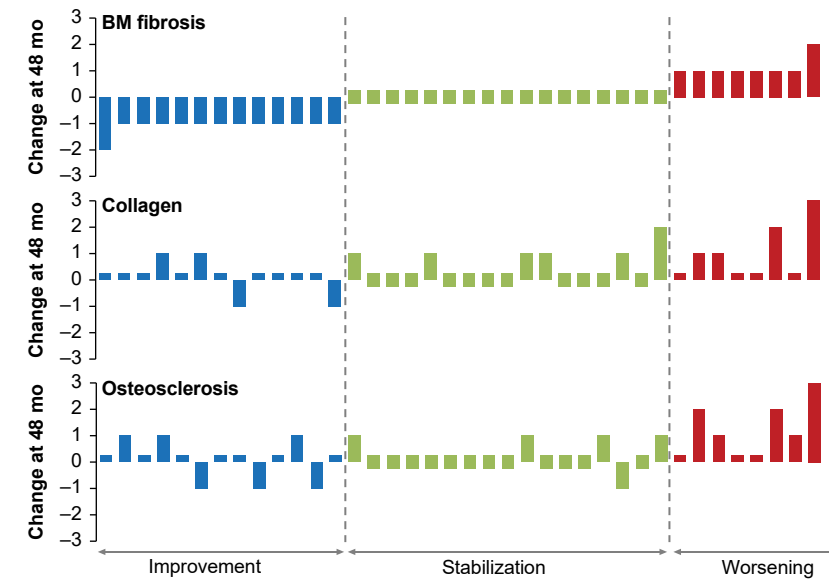

C. 60 Months

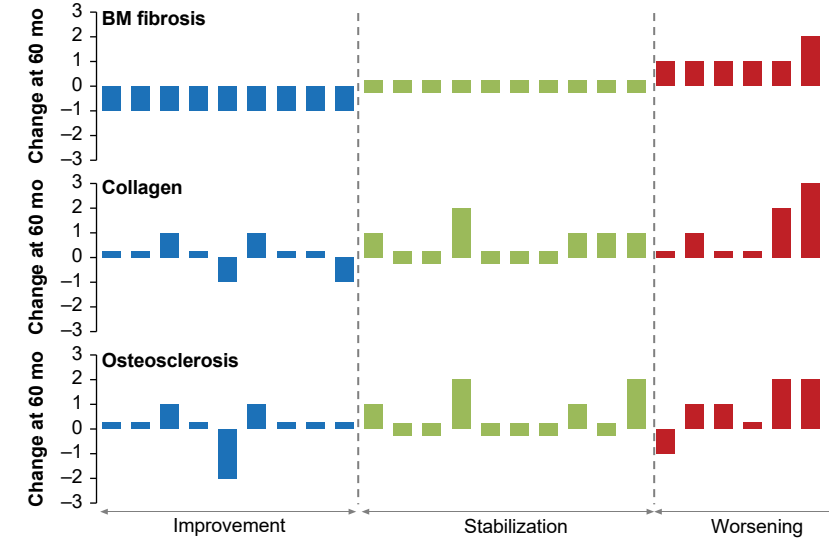

Supplement: Supplementary file 1 — Figure S1. Changes in bone marrow reticulin fibrosis, collagen deposition, and osteosclerosis in individual patients on ruxolitinib therapy. (A) 24, (B) 48, and (C) 60 months. (PDF 180 kb) [file 13045_2018_585_MOESM1_ESM.pdf]
